# Supplementary material for: Optimal path planning of Unmanned Aerial Vehicles (UAVs) for targets touring: Geometric and arc parameterization approaches
Source: PLoS One. 2022 Oct 14;17(10):e0276105. doi: 10.1371/journal.pone.0276105 (PMC9565756; doi:10.1371/journal.pone.0276105)
Supplement: Attachment — Submitted filename: Response to Reviewers.pdf [file pone.0276105.s001.pdf]

Detailed Response to Reviewers for

“Optimal Path Planning of Unmanned Aerial Vehicles (UAVs) for Targets Touring: Geometric and Arc Parameterization Approaches”

M. Forkan *

M. M. Rizvi †

M. A. M. Chowdhury‡

August 6, 2022

*Department of Mathematics, University of Chittagong, Chittagong-4331, Bangladesh, email: forkan.math@cu.ac.bd,

†Department of Mathematics, University of Chittagong, Chittagong-4331, Bangladesh email: mmrizvi@cu.ac.bd (corresponding author),

‡JNIRCMPS, University of Chittagong, Chittagong-4331, Bangladesh, email: mamansur@cu.ac.bd

Dear Editor,

We thank the editor and reviewers for their detailed and useful comments that helped us improve our manuscript significantly. We also thank the editor for giving us the opportunity to revise our work.

In what follows, we address each of the editor and referees comments, and explain how we incorporated the changes, which are highlighted in blue (the new text) in the revised manuscript.

Additional Editor Comments

Comment 1.1 Perform a comparison with other similar methods of literature.

Response. We have incorporated the Editor's suggestions and conducted further experiments to compare our proposed model (26) of the manuscript with the model given in [1, Section 5.1, Problem (Ps)].

We have included the following experimental results on Page 13 with slight adjustments in the revised manuscript.

We now compare our proposed model with the model given in [1, Section 5.1, Problem (Ps)]. In our comparison, we consider the single target touring problem and the comparison description is given in Example 3.

Example 3

We recall now Example 1 with an initial angle $\pi/3$. Set starting point $P_0(0, 0, \pi/3)$ and destination point $P_f(20, 8, \pi/6)$. The task is given to UAV to tour target $T(13, 0)$ with a minimum path chosen from (11). It is assumed that the minimum radius of the turning circle for UAV is $r = 2$, and UAV also follows the same radius circle when it intercepts the target and moves towards its destination P_f.

Results obtained for both models (26) and [1, Section 5.1, Problem (Ps)] demonstrated in Figures 1 and 2. It is shown that $RSLSR$ path is the optimum path to accomplish the task. In addition, subarc lengths of optimal solutions are provided in Table 1 for both models.

In our analysis, we observe that both models produce the similar kind of minimum path. We found that the total length of the path obtained by the models (26) and [1, Section 5.1,

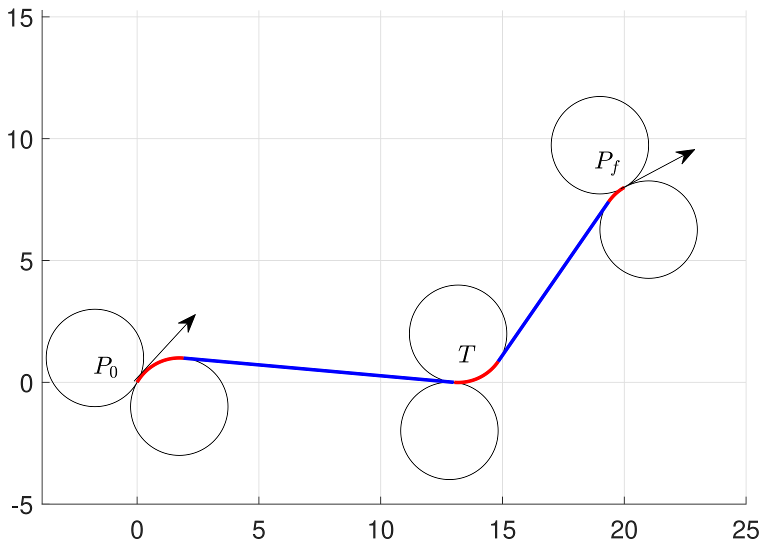

Figure 1: Shortest path generated by the model (26).

Problem (Ps)] is 24.30 and 24.13, respectively. Note that the computational time taken by the solver [39] is 0.125 seconds, the total time elapsed by the system 0.155 seconds, and the simulation time taken by MATLAB is approximately 1.5 seconds..

Table 1: *Numerical performance of model (26) and [1, Section 5.1, Problem (Ps)].*

Models	Left turn L_{l_1}	Right turn L_{l_2}	Straight line L_{l_3}	Left turn L_{l_4}	Right turn L_{l_5}	Straight line L_{l_6}	Left turn L_{l_7}	Right turn L_{l_8}
Problem (26)	0	2.17	11.13	2.11	0	7.96	0	0.89
Problem [1, (Ps)]	0	2.32	10.13	1	0	8.95	0	0.73

□

Comment 1.2 Report the computation time for the simulations.

Response. Thank you for pointing this out. Our experiments are two-fold. We first model the problem in AMPL and solve the problem using efficient solvers which has been reported in the manuscript. Once we get the solutions, we use MATLAB to visualize the output. Therefore, in the revised version, we included the total time elapsed by the solver, total time taken by the system and total visualization time for each of our experiments. The

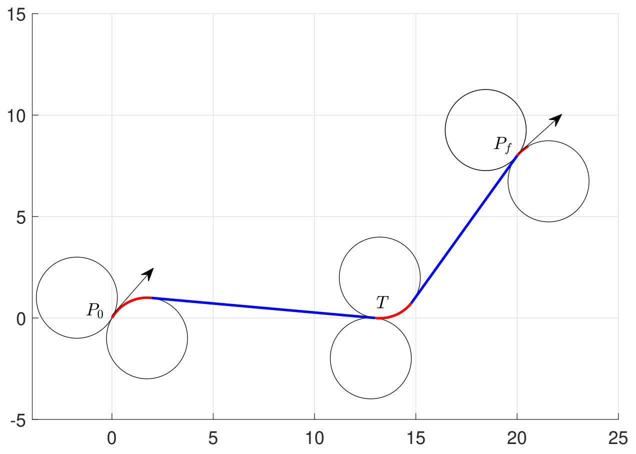

Figure 2: Shortest path generated by the model [1, Section 5.1, Problem (Ps)].

computational time for examples can be seen in the 'revised manuscript' by pointing to newly added texts in blue color. □

Comment 1.3 Report the memory consumption for each simulation.

Response. In our analysis, the computations have been performed on a Dell Inspiron laptop with 16 GB RAM and core i7 at 4.6 GHz. Each simulation of our analysis requires memory consumption of 29228 MB on average. As per suggestion, we have now reported memory consumption information in the revised manuscript on Page 14.

□

Comment 1.4 Please validate your proposal with a real robot.

Response.

Our analysis related to obtaining the shortest path in $2D$ environment with the simulation demonstrated widely depends on the 2-dimensional coordinates system. To make some tests in the real world and in our current simulation to validate the usefulness of our work in real robot, we need to extend the current analysis in $3D$ coordinate system that requires theoretical and algorithmic development. We appreciate Editor's suggestion and keep this implementation as our future research work. □

Comment 1.5 Please, extend the explanation about how you will deal with obstacle avoidance in a future work, give more details.

Response. We have incorporated the reviewer's suggestions and described how our proposed model could be extended to obstacle avoidance.

We have included the following clarifications on Page 15 with slight adjustments in the revised manuscript.

The material is presented in this paper can be extended for the obstacle avoidance problem. UAVs fly through obstacles such as buildings, hills, restricted zones, etc., which intercept the normal flight paths of the UAV. Sensors monitoring the UAV's environment for fixed or moving obstacles are usually used for obstacle avoidance. The obstacle avoidance problem is closely associated with path planning because obstacles typically result in the re-planning of paths. For obstacle avoidance, our proposed model requires slight changes in the geometrical approach given in Fig (1) and then requires expressing the mathematical functions to construct the flying paths. For example, suppose the obstacle is circular, in that case, the target point T must be on the circumference of the minimum turning circle centred at C (see Fig 3). The point T is variable here, and it is required to find T in such a way so that the flying path is minimum.

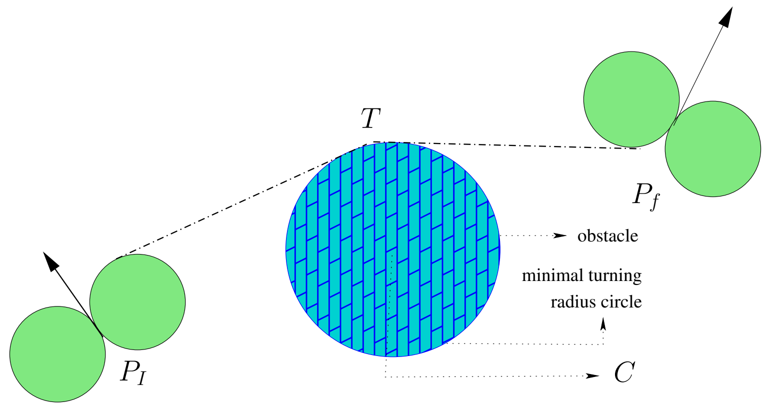

Figure 3: Circular obstacles.

□

Reviewer 1 Comments

Comment 2.6 Figure 1 and its description should be explained in a little more detail since the interpretation is confusing.

Response. We have incorporated the reviewer's suggestions and described Fig 1 in further detail.

We have included the following clarifications on Page 3 with slight adjustments in the revised manuscript.

Geometrical Interpretation Path Planning for Single Target Touring

This section illustrates path planning for UAV that start from an initial location, passes through a known target, and reach the finishing point. The proposed geometric approach describes an essential criterion for shortest route planning subject to the conditions that UAV tour the target. Our proposed geometrical interpretation of a feasible optimal path of type $CSCSC$ for a single target is the concatenation of two sub-paths of type CS and CSC. These sub-paths characterized by Dubins' path (Theorem 1, [8]) are demonstrated in Fig 1 in $2D$ environment. The complete methodology of constructing the paths for a UAV with a single target touring is given below.

In Fig 1, there is a target fixed at T. The UAV's starting and finishing points are P_I and P_f , respectively. X_i, $i = 1, \ldots, 8$ are set to calculate CS and CSC curves. For instance, the curve CS is calculated adding arc $P_I X_1$ and straight line $X_1 T$ if UAV turns left, and $P_I X_2 + X_2 T$ if it turns right in 2D environment. In Step-I of eqation describes how to approximate CS paths. The curve CSC is constructed as either any of paths presented in Step-II.

It is noted that initial configuration (a configuration consists of a position and a heading angle) $P_I(x_0, y_0, \psi_0)$ and the final configuration is $P_f(x_f, y_f, \psi_f)$. The fixed target location is $T(x_T, y_T, \psi_T)$, which is known in advance where ψ_T is the angle at T formed by straight lines $X_1 T$ or $X_2 T$ presented in (1). According to the Fig (1), UAV moves from P_I with a given heading angle ψ_0 and then flies towards target T, and after touring T, it turns along its destination P_f.

We divide the minimum path design for target touring problems into following two steps, as shown in Fig (1).

□

Comment 2.7 Equation 3 should be revised.

Response. We have incorporated the reviewer's suggestions. Now Equation (3) of old manuscript has been revised and referred to as Problem (P).

We have included the following clarifications on Page 5 with slight adjustments in the revised manuscript.

(P): Minimize (3), subject to the conditions are $\gamma(0) = P_I$, $\gamma(t_f) = P_f$, which are initial and destination points, respectively, velocities at initial and end points are $\dot{\gamma}(0) = v_0$, $\dot{\gamma}(t_f) = v_f$, respectively. Moreover,

$$||\ddot{\gamma}(t)|| \leq \alpha, ||\dot{\gamma}(t)|| = 1, \text{for a.e} \;\; t \in [0, t_f],$$

where $||v_0|| = ||v_f|| = 1$. □

Comment 2.8 Equations 5, 6, 7, 8, and 9 are not written in a standard math text. Review notation for "sin, arg, cos, for all".

Response. We thank the reviewer for pointing this out. We have rewritten Equations (5), (6), (7), (8), and (9) and used standard math text. We also used proper notations to write "sin, arg, cos, for all" which can be seen on Page 6 of the revised manuscript. □

Comment 2.9 The authors can enrich the explanation of section 4 using algorithms or flow charts.

Response. We have incorporated the reviewer's suggestions. In addition, we have included an algorithm in Section 4 to explain the numerical steps of our implementation.

We have included the following algorithm on Page 7 with slight adjustments in the revised manuscript.

Now we adopt mathematical approaches to construct the model for finding the optimal path of UAV with single target touring describe as follows.

Algorithm (Shortest Path)

Step 1 (Input)

Set left-turn arc ℓ_i, $i = 1, 4, 7$ right-turn arc ℓ_i, $i = 2, 5, 8$, and st. line ℓ_i, $i = 3, 6$.

Assume $\ell_i \neq 0$, $i = 3, 6$.

Step 2 **(Determine the length from initial point to target point)**

Set Curve-St.Line := CS.

Find $CS := \ell_1 + \ell_2 + \ell_3$ such that

Turns left:

$\ell_2 = 0$ and $CS := \ell_1 + \ell_3$,

Or

Turns right:

$\ell_1 = 0$ and $CS := \ell_2 + \ell_3$.

Step 3 **(Determine the length from target point to finishing point)**

Set Curve-St.Line-Curve := CSC

Find $CSC := \ell_4 + \ell_5 + \ell_6 + \ell_7 + \ell_8$ such that

Turns left-st-left:

$\ell_5 = 0$, $\ell_8 = 0$ and $CSC := \ell_4 + \ell_6 + \ell_7$,

Or

Turns left-st-right:

$\ell_5 = 0$, $\ell_7 = 0$ and $CSC := \ell_4 + \ell_6 + \ell_8$,

Or

Turns right-st-left:

$\ell_4 = 0$, $\ell_8 = 0$ and $CSC := \ell_5 + \ell_6 + \ell_7$,

Or

Turns right-st-right:

$\ell_4 = 0$, $\ell_7 = 0$ and $CSC := \ell_5 + \ell_6 + \ell_8$.

Step 3 Find optimal path that solves Problem (P):= $\min(CS + CSC)$.

To determine optimal path for single target touring, we combine the feasible paths obtained in Steps 2 and 3 and thus optimal path be one of the feasible paths listed in (11). For instance, if we obtain optimal of type $RSLSR$, then we attained lengths with $l_1 = l_5 = l_7 = 0$ and $l_2, l_3, l_4, l_6, l_8 > 0$.

□

Comment 2.10 The results of Section 5 should be contrasted with the results of similar

works. In addition, it is essential that the authors place the computation time and the amount of memory spend to obtain each result.

Response. We have incorporated the reviewer's suggestions and conducted further experiments to compare our proposed model (26) of the manuscript with the model given in [1, Section 5.1, Problem (Ps)].

We have included the experimental results on Page 13. The newly added texts can also be seen in Comment 1.1.

Regarding computational time: Thank you for pointing this out. Our experiments are two-fold. We first model the problem in AMPL and solve the problem using efficient solvers which has been reported in the manuscript. Once we get the solutions, we use MATLAB to visualize the output. Thus, in the revised version, we have included the total time elapsed by the solver, total time taken by the system and total visualization time for each of our experiments. The computational time for examples can be seen in the 'revised manuscript' by pointing to the newly added text in blue colour.

Memory consumption: In our analysis, the computations have been performed on a Dell Inspiron laptop with 16 GB RAM and core i7 at 4.6 GHz. Each simulation of our analysis requires memory consumption of 29228 MB on average. Therefore, we have also included memory consumption information in the revised manuscript on Page 14.

□

Reviewer 2

Comment 3.1 The paper is interesting, and presents a congruent mathematical analysis. I consider that the use of the drone at the same height should be justified in a solid way, as this is a point of improvement to the proposed analysis. I also consider that more experiments with radii of circumferences smaller than 2 should be shown.

Response.

We appreciate the reviewer's valuable comments and would like to thank you for finding our analysis interesting. In our analysis, we use $2D$ path and propose a new model to find the solution to the arc lengths so that we can identify the shortest path from the possible feasible solutions described by Dubin's [1] (reference of the revised manuscript).

We have now conducted more experiments which can be seen in Comment 1.1. We also conducted experiments taking smaller radii, and the following experiment has now been included with slight adjustment on Page 11 of the revised manuscript.

We also solved Example 2 considering with smaller radii $r = 1$, and obtained the shortest path depicted in Figure 4. Optimal solutions for the arc lengths are included in Table 2. The total length obtained by the algorithm is 44.85, and the computational time taken by the solver (Bonmin [41]) is 0.0625 seconds, the total time elapsed by the system 0.078 seconds, and the simulation time taken by MATLAB is 2 seconds.

Table 2: *Multiple Targets Touring – Numerical performance of model (26).*

Targets /lengths	Left turn L_{l_1}	Right turn L_{l_2}	Straight line L_{l_3}	Left turn L_{l_4}	Right turn L_{l_5}	Straight line L_{l_6}	Left turn L_{l_7}	Right turn L_{l_8}	Straight line L_{l_6}	Left turn L_{l_7}	Right turn L_{l_8}
T_1 & T_2 $r = 2$	0	1.66	12.65	0	2.27	13.96	3.68	0	10.01	0	1.84
T_1 & T_2 $r = 1$	0	0.81	13.42	0	1.12	14.90	1.66	0	12.16	0	0.78

□

References

[1] C. Y. Kaya (2019) Markov–Dubins interpolating curves, Computational Optimization and Application, 73(2) 647–677.

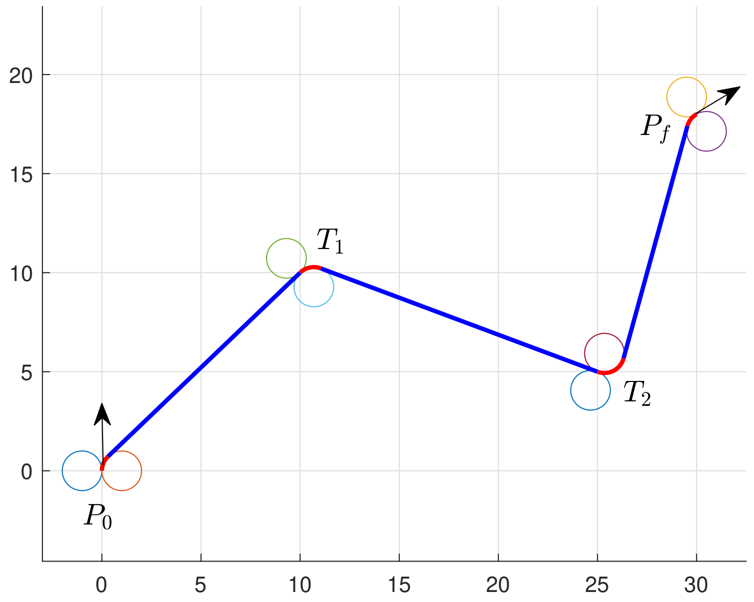

Figure 4: Minimum Path approximated for Example 2 when $r = 1$.
