# Supplementary material for: Optimal path planning of Unmanned Aerial Vehicles (UAVs) for targets touring: Geometric and arc parameterization approaches
Source: PLoS One. 2022 Oct 14;17(10):e0276105. doi: 10.1371/journal.pone.0276105 (PMC9565756; doi:10.1371/journal.pone.0276105)
Supplement: Attachment — Submitted filename: Response to Reviewers.pdf [file pone.0276105.s002.pdf]

Detailed Response to Reviewers for

"Optimal Path Planning of Unmanned Aerial Vehicles (UAVs) for Targets Touring: Geometric and Arc Parameterization Approaches"

M. Forkan *

M. M. Rizvi †

M. A. M. Chowdhury‡

September 18, 2022

*Department of Mathematics, University of Chittagong, Chittagong-4331, Bangladesh, email: forkan.math@cu.ac.bd,

†Department of Mathematics, University of Chittagong, Chittagong-4331, Bangladesh email: mmrizvi@cu.ac.bd (corresponding author),

‡JNIRCMPS, University of Chittagong, Chittagong-4331, Bangladesh, email: mamansur@cu.ac.bd

Dear Editor,

We thank the reviewers for their detailed and useful comments that helped us improve our manuscript significantly. We also thank the editor for giving us the opportunity to revise our work.

In what follows, we address reviewers comments, and explain how we incorporated the changes, which are highlighted in blue (the new text) in the revised manuscript.

Reviewer 1 Comments

We are pleased to know that Reviewer 1 has agreed with our responses. Thank you so much.

Comment 1.1 Some mathematical expressions in section 3 are not numbered.

Response. We have incorporated the reviewer's suggestions, and the mathematical expressions in Section 3 are numbered and referenced in the text. □

Comment 1.2 The comparison between the present work and similar ones is not clear, it is recommended to extend it in the discussion. Additionally, it is recommended to compare with more recent works less than 7 years old. If there are no recent works with which to compare, it is because the topic is not of interest, or what is the cause?

Response. We have incorporated the reviewer previous suggestions and conducted further experiments to compare our proposed model (30) of the manuscript with the model given at Problem (Ps) in Section 5.1 of following recent work.

[9] C. Y. Kaya (2019) Markov–Dubins interpolating curves, Computational Optimization and Application, 73(2) 647–677.

We apologise for confusing the reviewer pointing the reference [1] in our previous response. Reference [1] points to the reference in the document prepared for 'response to the reviewers'. However, this was the reference [9] of the revised manuscript.

We have incorporated the reviewer's suggestions and included the extended comparison through the following remark on Page 14 with adjusted Table reference.

Remark: The proposed model (30) provides the optimal path of UAV, travels from starting point to destination with a target touring, is partitioned through 8 segments (circular(C) or

straight line(S)). The concatenation of these segments is as follows. The lengths L_{l_1} and L_{l_2} (see Table 1 (Table 3 in revised manuscript)) are considered for the segments of left-turn and right-turn respectively at the initial point when the UAV approaches to target. The length L_{l_3} for the straight line segment joining the terminal point of circular subarc L_{l_1} or L_{l_2} and the target point, and the length of the consecutive segment are L_{l_4} and L_{l_5} of the left-turn and right-turn respectively, when UAV is headed towards its destination from the target point; similarly, three more lengths (L_{l_6}, L_{l_7} and L_{l_8}) of segments are required to reach the destination point. This is demonstrated in Table 1 (Table 3 in revised manuscript). On the other hand, the model [9, Section 5.1, Problem (Ps)] provides a concatenation of 10 segments with approximated lengths to obtain the optimal path for the same single target touring as mentioned above. In this case, the two segments $\hat{L}_{l_4}$ and $\hat{L}_{l_5}$ (see Table 1 (Table 3 in revised manuscript)) of total 10 segments are additionally appeared. Moreover, for n targets touring, model [9, Section 5.1, Problem (Ps)] requires $5n+5$ segments, whereas the proposed model (30) requires $3n+5$ segments with approximated lengths. As a result, there are need more $((5n+5)-(3n+5))$ or $2n$ segments to calculate the optimal path for n targets touring in the model [9, Section 5.1, Problem (Ps)] than that of the segments in the model (30). This difference is reflected in our numerical experiments and we have verified this for a single target touring. The computational time taken by the solver (Ipopt [39]) are 0.125 seconds and 0.135 seconds, and the total time elapsed by the system 0.155 seconds and 0.167 seconds, for models (30) and [9, Section 5.1, Problem (Ps)], respectively. Therefore, the proposed model might have a computational advantage over the model stated in [9].

Table 1: *Numerical performance of model (30) and [9, Section 5.1, Problem (Ps)].*

Models	Left turn L_{l_1}	Right turn L_{l_2}	Straight line L_{l_3}	Left turn L_{l_4}	Right turn L_{l_5}	Left Step $\hat{L}_{l_4}$	Right Step $\hat{L}_{l_5}$	Straight line L_{l_6}	Left turn L_{l_7}	Right turn L_{l_8}
Problem (30)	0	2.17	11.13	2.11	0	...	...	7.96	0	0.89
[9, Problem (Ps)]	0	2.32	10.13	1	0	1	0	8.95	0	0.73

□

Comment 1.3 The authors report a memory consumption of 29228 MB, is this the data stored during the simulation in ROM (Hard Disk) memory?

Response. Thank you for pointing this out. We reported combined RAM for AMPL and MATLAB during the simulation. We have now added the following information on Page 16.

Each simulation of our analysis requires memory consumption of 29228 MB (combined RAM for AMPL and MATLAB during simulation) on average. □

Reviewer 2

Thank you so much. We are delighted to know that Reviewer 2 has accepted our responses.
